# Partial agonism improves the anti-hyperglycaemic efficacy of an oxyntomodulin-derived GLP-1R/GCGR co-agonist

**DOI:** 10.1016/j.molmet.2021.101242

**Published:** 2021-04-30

**Authors:** Phil Pickford, Maria Lucey, Roxana-Maria Rujan, Emma Rose McGlone, Stavroula Bitsi, Fiona B. Ashford, Ivan R. Corrêa, David J. Hodson, Alejandra Tomas, Giuseppe Deganutti, Christopher A. Reynolds, Bryn M. Owen, Tricia M. Tan, James Minnion, Ben Jones, Stephen R. Bloom

**Affiliations:** 1 Section of Endocrinology and Investigative Medicine, Department of Metabolism, Digestion, and Reproduction, Imperial College London, London, W12 0NN, UK; 2 Centre for Sport, Exercise, and Life Sciences, Faculty of Health and Life Sciences, Coventry University, Alison Gingell Building, CV1 5FB, UK; 3 Section of Cell Biology and Functional Genomics, Department of Metabolism, Digestion, and Reproduction, Imperial College London, London, W12 0NN, UK; 4 Institute of Metabolism and Systems Research (IMSR) and Centre of Membrane Proteins and Receptors (COMPARE), University of Birmingham, Birmingham, UK; 5 Centre for Endocrinology, Diabetes and Metabolism, Birmingham Health Partners, Birmingham, UK; 6 New England Biolabs, Ipswich, MA, USA; 7 School of Life Sciences, University of Essex, Wivenhoe Park, Colchester, CO4 3SQ, UK

**Keywords:** GLP-1, Glucagon, Oxyntomodulin, Biased agonism, Partial agonism, β-arrestin

## Abstract

**Objective:**

Glucagon-like peptide-1 and glucagon receptor (GLP-1R/GCGR) co-agonism can maximise weight loss and improve glycaemic control in type 2 diabetes and obesity. In this study, we investigated the cellular and metabolic effects of modulating the balance between G protein and β-arrestin-2 recruitment at GLP-1R and GCGR using oxyntomodulin (OXM)-derived co-agonists. This strategy has been previously shown to improve the duration of action of GLP-1R mono-agonists by reducing target desensitisation and downregulation.

**Methods:**

Dipeptidyl dipeptidase-4 (DPP-4)-resistant OXM analogues were generated and assessed for a variety of cellular readouts. Molecular dynamic simulations were used to gain insights into the molecular interactions involved. In vivo studies were performed in mice to identify the effects on glucose homeostasis and weight loss.

**Results:**

Ligand-specific reductions in β-arrestin-2 recruitment were associated with slower GLP-1R internalisation and prolonged glucose-lowering action in vivo. The putative benefits of GCGR agonism were retained, with equivalent weight loss compared to the GLP-1R mono-agonist liraglutide despite a lesser degree of food intake suppression. The compounds tested showed only a minor degree of biased agonism between G protein and β-arrestin-2 recruitment at both receptors and were best classified as partial agonists for the two pathways measured.

**Conclusions:**

Diminishing β-arrestin-2 recruitment may be an effective way to increase the therapeutic efficacy of GLP-1R/GCGR co-agonists. These benefits can be achieved by partial rather than biased agonism.

## Highlights

• DPP-4-resistance-conferring amino acid substitutions affect pharmacological efficacy.
• GLP-1R/GCGR co-agonists with reduced β-arrestin recruitment are more effective in vivo.
• Partial agonism, rather than biased agonism, is sufficient to produce these benefits.

## Abbreviations

AIB: 2-aminoisobutyric acid
βarr: β-arrestin
cAMP: Cyclic adenosine monophosphate
DERET: Diffusion-enhanced resonance energy transfer
DPP-4: Dipeptidyl dipeptidase-4
ECL: Extracellular loop
GCG(R): Glucagon (receptor)
GIP(R): Glucose-dependent insulinotropic polypeptide (receptor)
GLP-1(R): Glucagon-like peptide-1 (receptor)
HCA: High content analysis
IPGTT: Intraperitoneal glucose tolerance test
OXM: Oxyntomodulin
PKA: Protein kinase A
T2D: Type 2 diabetes
TM: Transmembrane (helix)

## 1 Introduction

Insulin and glucagon are traditionally viewed as opposing protagonists in the hormonal control of blood glucose. Pharmacological approaches to potentiate glucose-stimulated insulin secretion (GSIS), such as analogues of the incretin glucagon-like pepide-1 (GLP-1), have been successfully exploited over many years to treat type 2 diabetes (T2D) [1]. However, decades of attempts to develop glucagon receptor (GCGR) antagonists for clinical use have thus far failed to yield any approved therapeutic agents [2]. A significant problem appears to be the development of hepatic steatosis [[3], [4], [5], [6]]. Contrasting with this traditional approach, GCGR agonism has emerged as a credible component of combined therapeutic strategies for treating obesity and T2D in which GLP-1R and GCGR are concurrently targeted [7,8], thereby recapitulating the effects of the endogenous GLP-1R/GCGR co-agonist oxyntomodulin (OXM) [9]. Well-established effects of glucagon on energy expenditure [10] leading to enhanced weight loss and ultimately improvements in insulin sensitivity [11] might be safely realised in the context of GLP-1R-mediated protection against acute hyperglycaemia. Glucagon is also insulinotropic, an effect that derives from action at both GLP-1R and GCGR [12,13].

Biased agonism is a concept in which different ligands for the same receptor selectively couple to different intracellular effectors [14], potentially providing a method of improving their therapeutic window by reducing the activation of pathways associated with adverse effects [15]. For G protein-coupled receptors (GPCRs), bias is commonly but not always expressed as a relative preference for recruitment of G proteins vs β-arrestins, that is, two of the most proximal interactors recruited to the activated receptor as well as their corresponding signalling intermediates. Both GLP-1R and GCGR are primarily coupled to cAMP generation through Gαs activation, with recruitment of β-arrestins being associated with receptor desensitisation, endocytosis, and diminished long-term functional responses [16,17]. While the therapeutic benefits of biased GLP-1R agonism have been demonstrated in a number of preclinical studies [[18], [19], [20], [21]], applying this principle to GLP-1R/GCGR co-agonism has been less explored. A recent study reported bias profiles for a selection of investigational dual GLP-1R/GCGR agonists, but it is not clear what role bias plays in their metabolic effects [22].

In this study, we aimed to develop GLP-1R/GCGR co-agonists with altered signalling properties but otherwise equivalent characteristics, which might be used to assess the functional impact of bias in vitro and in vivo. Focussing on the peptide N-terminus, we evaluated dipeptidyl dipeptidase-4 (DPP-4)-resistant peptides featuring 2-aminoisobutyric acid (AIB) at position 2 in combination with which a switch between glutamine (Q) and histidine (H) at position 3 was able to alter the maximum responses (that is, efficacy) for G protein and β-arrestin recruitment to varying degrees at both receptors. Molecular dynamics simulation of glucagon analogues interacting with GCGR was used to gain insight into the molecular interactions underlying these differences. By comparing the metabolic effects of a pair of matched peptides with these sequence substitutions, we demonstrate that reduced recruitment efficacy of β-arrestins translates into improved efficacy in preclinical rodent models of obesity, consistent with a similar effect previously observed for GLP-1R mono-agonists [[18], [19], [20], [21]]. Our study therefore suggests a viable strategy to optimise GLP-1R/GCGR co-agonism for enhanced therapeutic efficacy.

## 2 Materials and methods

### 2.1 Peptides

All of the peptides were obtained from Wuxi Apptec and were at least 90% pure.

### 2.2 Cell culture

HEK293T cells were maintained in DMEM supplemented with 10% FBS and 1% penicillin/streptomycin. PathHunter CHO–K1-βarr2-EA cells stably expressing human GLP-1R, GCGR, or GIPR and PathHunter CHO–K1-βarr1-EA cells stably expressing GCGR were obtained from DiscoverX and maintained in Ham's F12 medium with 10% FBS and 1% penicillin/streptomycin. Stable HEK293-SNAP-GLP-1R cells [23] were maintained in DMEM supplemented with 10% FBS, 1% penicillin/streptomycin, and 1 mg/ml of G418. INS-1 832/3 cells, a gift from Professor Chris Newgard (Duke University) [24], were maintained in RPMI with 11 mM of glucose supplemented with 10% FBS, 10 mM of HEPES, 1 mM of pyruvate, 50 μM of β-mercaptoethanol, and 1% penicillin/streptomycin. Huh7 cells stably expressing human GCGR (Huh7-GCGR) [25] were maintained in DMEM supplemented with 10% FBS, 1 mg/ml of G418, and 1% penicillin/streptomycin.

### 2.3 Animal husbandry

The animals were maintained in specific pathogen-free facilities, with ad libitum access to food (except prior to fasting studies) and water. The studies were regulated by the UK Animals (Scientific Procedures) Act 1986 of the UK and approved by the Animal Welfare and Ethical Review Body of Imperial College London (Personal Project License PB7CFFE7A) or the University of Birmingham (Personal Project License P2ABC3A83). Specific procedures are described as follows.

### 2.4 Primary islet isolation and culture

The mice were euthanised by cervical dislocation before injection of collagenase solution (1 mg/ml of Serva NB8 or S1745602, Nordmark Biochemicals) into the bile duct. Dissected pancreata were then digested for 12 min at 37 °C in a water bath before purification of islets using a Ficoll (1.078) or Histopaque (Histopaque-1119 and -1083) gradient. Islets were hand-picked and cultured (5% CO2, 37 °C) in RPMI medium containing 10% FBS and 1% penicillin/streptomycin.

### 2.5 Primary hepatocyte isolation and culture

Hepatocytes from adult male C57Bl/6J mice were isolated using collagenase perfusion [26]. After filtering and washing, the cells were used to directly assay cAMP responses as described in the next section.

### 2.6 NanoBiT assays and calculation of bias between mini-Gs and β-arrestin-2

The assay was performed as previously described [21]. HEK293T cells in 12-well plates were transfected with 0.5 μg of GLP-1R-SmBiT plus 0.5 μg of LgBiT-mini-Gs, -mini-Gi, or -mini-Gq [27] (gifts from Professor Nevin Lambert, Medical College of Georgia) or 0.05 μg of GLP-1R-SmBiT and 0.05 μg of LgBiT-β-arrestin-2 (Promega) plus 0.9 μg of pcDNA3.1 for 24 h. The cells were detached with EDTA, resuspended in HBSS, and furimazine (Promega) was added at a 1:50 dilution from the manufacturer's pre-prepared stock. After dispensing into 96-well white plates, a baseline read of the luminescent signals was serially recorded for 5 min using a FlexStation 3 instrument at 37 °C before the indicated concentration of ligand was added, after which the signals were repeatedly recorded for 30 min. For AUC analysis, the results were expressed relative to the individual well baseline for AUC calculations over the 30-min stimulation period. Baseline drift over time frequently led to a negative AUC for vehicle treatment, which was subtracted from all of the results before construction of 3-parameter curve fits of the concentration-response using Prism 8.0. Bias was calculated using two approaches. First, the log max/EC50 method [28] was used, with the ratio of Emax to EC50 from 3-parameter fits for each ligand used to quantify agonism. After log10 transformation, responses were expressed relative to the reference agonist on a per assay basis to obtain Δlog(Emax/EC50) for each pathway. Pathway-specific values were then expressed relative to each other to obtain ΔΔlog(Emax/EC50), that is, the log bias factor. Second, a method derived from kinetic curve fitting was used [29]. Here, kinetic responses for a single maximal agonist concentration were normalised at each time point to the vehicle response prior to curve fitting. Mini-Gs responses were fitted using the one-phase exponential association equation in Prism 8.0. β-arrestin-2 responses were fitted using the biexponential equation described in [29]. The agonist efficacy term kτ was derived from these data as described [29] for each agonist and, after log10 transform, the SRB103Q response was expressed relative to SRB103H as the reference agonist on a per assay basis to obtain Δlog kτ. Pathway-specific values were then expressed relative to each other to obtain ΔΔlog kτ, that is, the log bias factor.

### 2.7 Biochemical measurement of cAMP production

PathHunter cells were stimulated with the indicated concentration of agonist for 30 min at 37 °C in serum-free medium without phosphodiesterase inhibitors. INS-1 832/3 cells were stimulated with the indicated concentration of agonist for 10 min at 37 °C in serum-free medium with 100 μM of 3-isobutyl-1-methylxanthine (IBMX). Primary dispersed mouse islet cells prepared by triturating intact islets in 0.05% trypsin/EDTA for 3 min at 37 °C were stimulated with the indicated concentration of agonist for 5 min at 37 °C in serum-free medium with 11 mM of glucose and 500 μM of IBMX. Primary mouse hepatocytes were stimulated in serum-free medium with 100 μM of IBMX for 10 min or 16 h in serum-free medium with 100 μM of IBMX added for the final 10 min of incubation. Huh7-GCGR cells were stimulated in serum-free medium without phosphodiesterase inhibitors for 10 min or for 16 h in serum-free medium. Where relevant, forskolin (10 μM) was added as a positive or normalisation control. At the end of each incubation, cAMP was then assayed by HTRF (Cisbio cAMP Dynamic 2) and concentration-response curves were constructed with 3-parameter curve fitting using Prism 8.0.

### 2.8 Dynamic cAMP imaging in intact islets

C57Bl/6 (n = 7) or Ins1tm1.1(cre)Thor+/- (n = 2) mice were used as islet donors and were phenotypically indistinguishable. Islets were transduced with epac2-camps for 48 h using an adenoviral vector (a gift from Professor Dermot M. Cooper, University of Cambridge). Epac2-camps is well validated, relatively pH insensitive, and senses cAMP concentrations in the ranges described in islets [30,31]. Dynamic cAMP imaging was performed as previously described [32] using a Crest X-Light spinning disk system coupled to a Nikon Ti-E microscope base and a 10 × objective. Excitation was delivered at *λ* = 430–450 nm using a SPECTRA X light engine. Emitted signals were detected using a 16-bit Photometrics Evolve Delta EM-CCD at *λ* = 460–500 nm and 520–550 nm for cerulean and citrine, respectively. For imaging, islets were maintained in HEPES-bicarbonate buffer (pH 7.4) containing (in mM): 120 NaCl, 4.8 KCl, 24 NaHCO3, 0.5 Na2HPO4, 5 HEPES, 2.5 CaCl2, 1.2 MgCl2, and 16.7 d-glucose. The experiment was conducted to determine responses to agonist-naïve islets (“acute”) or with a “rechallenge” design in which islets were first treated for 4 h with 100 nM of agonist followed by washout (2 washes for 30 min) before imaging. During imaging, the islets were stimulated with 100 nM of agonist for 15 min starting at T = 5 min, followed by application of 10 μM of forskolin as a positive control. FRET responses were calculated as the fluorescence ratio of cerulean/citrine and normalised as F/F0-5, where F denotes the fluorescence at any given time point and F0-5 denotes the average fluorescence for 0–5 min.

### 2.9 High content imaging assay for receptor internalisation

The assay was adapted from a previously described method [33]. HEK293T cells were seeded in black clear-bottom plates coated with 0.1% poly-d-lysine and assayed 24 h after transfection with SNAP-tagged GLP-1R or GCGR plasmid DNA (0.1 μg per well). The cells were labelled with the cleavable SNAP-tag probe BG-S-S-549 (a gift from New England Biolabs) in complete medium for 30 min at room temperature. After washing, fresh serum-free medium ± agonist was added. At the end of incubation, the medium was removed and the wells were treated with for 5 min at 4 °C with Mesna (100 mM in alkaline TNE buffer at a pH of 8.6) to remove BG-S-S-549 bound to residual surface receptors without affecting the internalised receptor population or with alkaline TNE buffer alone. After washing, phase contrast and epifluorescence cellular imaging at 20× magnification was performed, followed by processing as previously described [33] to quantify the amount of internalised receptor from the fluorescence intensity readings.

### 2.10 High content imaging for fluorescent ligand internalisation

Huh7-GCGR cells were seeded in black clear-bottom plates coated with 0.1% poly-d-lysine and assayed 24 h later. Then 100 nM of fluorescent TMR-conjugated agonist or vehicle was applied for 30 min. The cells were then washed with cold HBSS and incubated for 5 min in cold acetic acid +150 mM of NaCl buffer at a pH of 2.9 to strip surface ligands. After a final wash, the cells were resuspended in HBSS and the fluorescent ligand uptake was measured and quantified by high content imaging as described in Section 2.9.

### 2.11 Imaging of fluorescent ligand uptake in pancreatic islets

Mouse pancreatic islets were isolated and left to recover overnight before being immobilised with Matrigel onto glass-bottom Mattek dishes and stimulated with 100 nM of the indicated TMR-modified agonist for 30 min. Z stacks were recorded for the whole islet volume on a Zeiss LSM780 inverted confocal microscope with a 20× air objective and 1 μm of separation between optical slices.

### 2.12 Preparing and imaging fixed cell samples to observe receptor internalisation

Cells were seeded onto coverslips coated with 0.1% poly-d-lysine and assayed 24 h after transfection with SNAP-tagged GLP-1R or GCGR plasmid DNA (0.5 μg per well of a 24-well plate). Surface labelling of the SNAP-tagged GLP-1R was performed using 0.5 μM of the indicated SNAP-surface probe for 30 min at 37 °C before washing with HBSS. Ligands were applied in Ham's F12 media at 37 °C. For fixation, 4% paraformaldehyde (PFA) was applied directly to the medium for 15 min before washing with PBS. Slides were mounted in Prolong Diamond antifade with DAPI and allowed to set overnight. Widefield epifluorescence imaging was performed using a Nikon Ti2E custom microscope platform via a 100×1.45 NA oil immersion objective, followed by Richardson-Lucy deconvolution using DeconvolutionLab2 [34].

### 2.13 Measuring GLP-1R internalisation using DERET

The assay was performed as previously described [21]. HEK-SNAP-GLP-1R cells were labelled using 40 nM of SNAP-Lumi4-Tb in complete medium for 60 min at room temperature. After washing, the cells were resuspended in HBSS containing 24 μM of fluorescein and dispensed into 96-well white plates. A baseline read was serially recorded for 5 min using a FlexStation 3 instrument at 37 °C in TR-FRET mode using the following settings: λex at 340 nm, λem at 520 and 620 nm, auto cut-off, delay of 400 μs, and integration time of 1500 μs? Ligands were then added, after which the signals were repeatedly recorded for 30 min. The fluorescence signals were expressed ratiometrically after first subtracting signals from wells containing 24 μM of fluorescein but no cells. Internalisation was quantified as the AUC relative to the individual well baseline, and concentration-response curves were generated with Prism 8.0.

### 2.14 Insulin secretion assay

INS-1 832/3 cells were seeded in suspension into complete medium with 11 mM of glucose ± agonist and incubated for 16 h at 37 °C. Secreted insulin in the supernatant was analysed by HTRF (Insulin High Range kit, Cisbio) after dilution and normalised to the concentration in glucose-only treated wells.

### 2.15 DPP-4 peptide degradation assay

A total of 10 nmol of SRB103Q, SRB103H, or GLP-1 was dissolved in 750 μl of DPP-4 buffer (100 mM of Tris–HCl at a pH of 8). Then, 10 mU of recombinant DPP-4 (R&D Systems) or no enzyme as a control for non-enzymatic degradation over the same time period was added to the reconstituted peptide. Reactions were incubated at 37 °C and 120 μl samples were collected from the reaction vessel at the indicated time points. Then, 5 μl of 10% trifluoroacetic acid (TFA) was added to each sample to terminate enzyme activity. The samples were analysed by reverse-phase high-performance liquid chromatography (HPLC) with a linear acetonitrile/water gradient acidified with 0.1% TFA on Phenomenex Aeris Peptide 3.6 μm XB-C18 columns (150 × 4.6 mm). The eluted peptides were detected at 214 nm. Peptide degradation was calculated by comparing the area under the peak of the original compound with and without enzyme.

### 2.16 In vivo studies

Lean male C57Bl/6 mice (8–10 weeks of age with a body weight of 25–30 g obtained from Charles River) were maintained at 21–23 °C and 12-h light–dark cycles. Ad libitum access to water and normal chow (RM1, Special Diet Services) or diet containing 60% fat to induce obesity and glucose intolerance (D12492, Research Diets) for a minimum of 3 months before experiments was provided. The mice were housed in groups of 4, except for food intake assessments and the chronic administration study, when they were individually caged with 1 week of acclimatisation prior to experiments. Treatments were randomly allocated to groups of mice matched for weight.

### 2.17 Intraperitoneal glucose tolerance tests

The mice were fasted for at least 4 h before commencing the glucose tolerance test depending on the peptide treatment length. Peptide or vehicle (0.9% saline) was injected into each mouse's intraperitoneal (IP) cavity either 8 h before, 4 h before, or at the same time as the glucose challenge (acute). Glucose was dosed at 2 g/kg of body weight. Blood glucose levels were measured before a glucose challenge then at the times as indicated in the figure using a hand-held glucose meter (GlucoRx Nexus). Blood samples for insulin were collected at 10 min into lithium heparin-coated microvette tubes (Sarstedt, Germany), followed by centrifugation (10,000 RPM for 8 min at 4 °C) to separate the plasma. Plasma insulin was measured using a Cisbio mouse insulin HTRF kit.

### 2.18 Insulin tolerance tests

The mice were fasted for 2 h before IP injection of peptide or vehicle (0.9% saline). Four h later, baseline blood glucose was taken before recombinant human insulin (Sigma, USA) (0.5 U/kg-1 U/kg) was IP injected and blood glucose was measured 20, 40, and 60 min after insulin injection.

### 2.19 Feeding studies

The mice were fasted overnight before the study. Diet was returned to the cage 30 min after IP injection of agonist, with cumulative intake determined by weighing.

### 2.20 Pharmacokinetic study

The mice were administered 0.5 mg/kg of peptide via IP injection. Four h after injection, blood was acquired by venesection into lithium heparin-coated microvette tubes (Sarstedt, Germany). In a separate study, male Sprague Dawley rats (average weight 250 g, obtained from Charles River) were administered 4 mg/kg of peptide SC mixed in aqueous ZnCl2 solution to a molar ratio of 0.7:1 (peptide:ZnCl2), and blood was collected by venesection at several time points up to 72 h. Plasma was separated by centrifugation at 10,000 g for 8 min at 4 °C. Plasma concentrations were assessed by radioimmunoassay using an in-house assay as previously described [35] using standard curves generated from each SRB103 peptide to ensure that equivalent recovery was obtained.

### 2.21 Chronic administration study

SRB103 peptides were mixed in aqueous ZnCl2 solution to a molar ratio of 1.2:1 (ZnCl2:peptide). Liraglutide (Novo Nordisk) was diluted in sterile water. DIO mice received daily subcutaneous (s.c.) injections of each treatment or vehicle (matched ZnCl2) with the dose increased during the first week as indicated in the figure. Body weight and food intake was measured periodically, with food and water available ad libitum. The end-of-study glucose tolerance test was performed 8 h after the final peptide dose with the mice fasted for 5 h. Body composition was measured by EchoMRI at the end of the study.

### 2.22 Statistical analysis of biological data

Quantitative data were analysed using Prism 8.0 (GraphPad Software). In cell culture experiments, technical replicates were averaged so that each individual experiment was treated as one biological replicate. Dose responses were analysed using 3- or 4-parameter logistic fits with constraints imposed as appropriate. Bias analyses were performed as described in Section 2.6. Statistical comparisons were made by t-tests or ANOVA as appropriate, with paired or matched designs used depending on the experimental design. Mean ± standard error of mean (SEM) with individual replicates in some cases are displayed throughout with the exception of bias analyses, for which 95% confidence intervals are shown to allow straightforward identification of biased ligands, for which the 95% confidence bands did not cross zero. Statistical significance was inferred if p < 0.05 without ascribing additional levels of significance.

### 2.23 Systems preparation, equilibration, and molecular dynamics simulation

We performed molecular dynamics simulations on the active GCGR structure in complex with peptides GCG, GCG-AIB2, and GCG-AIB2H3 and the C-terminal helix 5 of the Gs protein's α-subunit. The structure was modelled using MODELLER software (https://salilab.org/modeller) [36]. The templates used were the full-length crystal structure of a partially activated GCGR in complex with NNC1702 peptide (PDB: 5YQZ) [37] and the cryo-EM structure of the active glucagon-like peptide-1 receptor (GLP-1R) (PDB: 6B3J) [38]. Maestro software (https://www.schrodinger.com/) was employed to add the missing residue H1 and substitute the adequate residues to generate GCG, GCG-AIB2, and GCG-AIB2H3. Once the three systems were complete and the hydrogens added, each system was embedded in a phospholipidic membrane and solvated. The membrane model used was 1-palmitoyl,2-oleoyl-sn-glycero-3-phosphochholine (POPC), which was generated by CHARMM-GUI (http://charmm-gui.org/). The simulation box dimensions of the resulting systems were 90 × 90 × 170 Å in the X, Y, and Z directions, respectively. General charge neutrality was obtained by adding Na+ and Cl-neutralising counter ions. Each system was subjected to 10,000 cycles of energy minimisation to eliminate steric clashes and relax the side chains. The final step before running the simulations was represented by the equilibration of the systems, which included re-orientations of the water and lipid molecules around the protein. The systems were both equilibrated and simulated in an NVT ensemble with semi-isotropic pressure scaling with a constant surface tension dynamic of 0 dyne/cm (through interfaces in the XY plane). The target pressure of 1 bar was achieved using the Monte Carlo barostat, while the target temperature of 300 K was regulated using Langevin dynamics with a collision frequency of 1 ps-1. The SHAKE algorithm was used to constrain the lengths of bonds comprising hydrogen atoms. Each system was equilibrated for 32 ns at a time step of 2 fs and then run in 3 replicas for approximately 2 μs at a time step of 4 fs using the AMBER force field implemented in the AMBER software package (http://ambermd.org/) [39].

### 2.24 MD trajectory analysis

Each replica of a system was merged and aligned on the initial frame using MDTraj (www.mdtraj.org/) and then analysed. The hydrogen bonds and van der Waals interactions between peptides and receptors were computed using the GetContacts package (https://getcontacts.github.io/). The contacts were plotted on the PDB coordinates using in-house scripts and Chimera software (www.cgl.ucsf.edu/). The distances between T3696.60 located at the top of TM6 and the origin of the cartesian coordinates (0, 0, 0) were quantified using the open-source community-developed library PLUMED 2.0 (www.plumed.org). Using the data provided by PLUMED, we further calculated the distances’ distribution via an in-house script. Principal component analysis (PCA) was conducted on Cα atoms using the R package Bio3D (www.thegrantlab.org/) [40]. Prior to PCA, we carried out a trajectory frame superposition on Cα atoms of residues 133 to 403 (TM domain) to minimise the root mean square differences among the equivalent residues. The principal component 1 (PC1) graphic representation was displayed through the Pymol Molecular Graphics System (https://pymol.org/).

## 3 Results

### 3.1 Characterising N-terminal peptide substitutions that modulate coupling to Gαs and β-arrestin-2 at GLP-1R and GCGR

**Figure 1:**
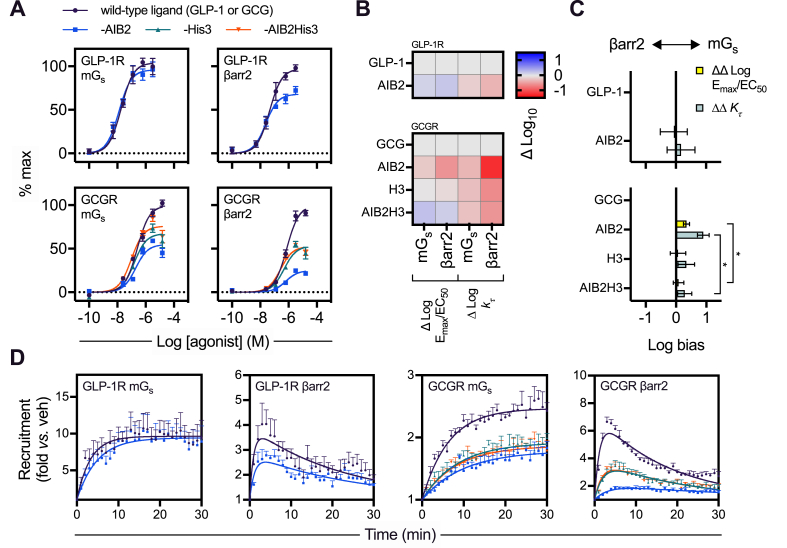
Evaluation of N-terminal substitutions to GLP-1, glucagon, or OXM. (A) Concentration responses with 3-parameter fits showing mini-Gs (mGs) or β-arrestin-2 (βarr2) recruitment to GLP-1R-SmBiT or GCGR-SmBiT in HEK293T cells stimulated with GLP-1, GLP-1-AIB2, glucagon (GCG), GCG-AIB2, GCG-H3, or GCG-AIB2H3, n = 5, with 3-parameter fits shown. (B) Heatmap representation of mean responses after quantification by log(max/EC50) or the kτ method and normalisation to the reference ligand (GLP-1 or GCG, as appropriate). (C) Assessment of bias between mini-Gs and β-arrestin-2 recruitment from log(max/EC50) or the kτ method, with statistical comparison by randomised block one-way ANOVA with Sidak's test comparing GCG-AIB2 and GCG-AIB2H3. The 95% confidence intervals are shown to allow identification of ligands with statistically significant bias vs the reference ligand. (D) Single maximal concentration kinetic responses of each ligand/receptor/pathway combination using the data shown in (A), with one-phase association fits for mini-Gs and bi-exponential fits for β-arrestin-2. ∗p < 0.05 indicated by the statistical test. Data are represented as mean ± SEM for concentration response curves or 95% confidence intervals for bias plots; bias data are considered significant if the 95% confidence interval does not cross 0.

The N-termini of GLP-1, glucagon, and OXM play critical roles in activating their target receptors [33,41]. However, alanine (in GLP-1) or serine (in glucagon and OXM) at position 2 renders each of these endogenous ligands susceptible to DPP-4-mediated cleavage, and pharmacologically stabilised incretin analogues are often modified at this position. In this study, we focussed on the AIB2 substitution found in semaglutide and some investigational oxyntomodulin analogues [[42], [43], [44]]. To systematically investigate how this change affects receptor activation, we obtained GLP-1-AIB2 and glucagon-AIB2 (GCG-AIB2) (see Table 1) and measured recruitment of β-arrestin-2 and mini-Gs to GLP-1R and GCGR in real time using nanoBiT complementation [27,45]. Area-under-curve (AUC) quantification from the kinetic response data indicated that efficacy for β-arrestin-2 recruitment to GLP-1R was modestly reduced with GLP-1-AIB2 compared to native GLP-1 (Table 2, Figure 1A, and Supplementary Fig. 1A). However, quantifying bias using the log(max/EC50) scale [28] indicated that this selective efficacy reduction did not qualify GLP-1-AIB2 as a biased agonist as it was compensated by a correspondingly small increase in potency (Figure 1B,C). The lack of bias is represented in Figure 1C by the 95% confidence intervals for GLP-1-AIB2 crossing zero. At GCGR, the impact of AIB2 was more striking, with large reductions in efficacy for both mini-Gs and β-arrestin-2 (Figure 1A); interestingly, this effect at GCGR could be partly reversed for both pathways by concurrent substitution of glutamine (Q) at position 3 to histidine (H), which our in-house preliminary evaluations had already flagged as a route to modulate GCGR signalling. GCG-AIB2 showed a moderate but statistically significant degree of bias in favour of mini-Gs recruitment, with the H3 substitution driving the bias factor back towards zero (Figure 1B,C).

**Table 1:** Amino acid sequences of the peptides used in this study. Amino acid sequences are given in single letter code. GLP-1 is amidated at the C-terminus as indicated. AIB is represented as X. Tetramethylrhodamine is indicated as TMR.

Peptide	Amino acid sequence
GLP-1	HAEGTFTSDVSSYLEGQAAKEFIAWLVKGR-NH2
GLP-1-AIB2	HXEGTFTSDVSSYLEGQAAKEFIAWLVKGR-NH2
GCG	HSQGTFTSDYSKYLDSRRAQDFVQWLMNT
GCG-AIB2	HXQGTFTSDYSKYLDSRRAQDFVQWLMNT
GCG-H3	HSHGTFTSDYSKYLDSRRAQDFVQWLMNT
GCG-AIB2H3	HXHGTFTSDYSKYLDSRRAQDFVQWLMNT
SRB103Q	HXQGTFTSDYSKYLDAKRAQEFIEWLLAGHHHHHPSW
SRB103H	HXHGTFTSDYSKYLDAKRAQEFIEWLLAGHHHHHPSW
SRB103Q-TMR	HXQGTFTSDYSKYLDAKRAQEFIEWLLAGHHHHHPS(K-TMR)W
SRB103H-TMR	HXHGTFTSDYSKYLDAKRAQEFIEWLLAGHHHHHPS(K-TMR)W

**Table 2:** Effect of AIB2 substitution in GLP-1, glucagon, or OXM on mini-Gs and β-arrestin-2 recruitment responses. Mean ± SEM parameter estimates from 3-parameter fitting of AUC data from Figure 1A and association rate constants at maximal agonist stimulation (K@[max]). Statistical comparisons performed by paired t-tests (GLP-1 and GLP-1-AIB2) or randomised block one-way ANOVA with Dunnett's test (glucagon analogues). Note that, in general, if > 1 ligand was a full agonist, Emax values were compared after normalisation to the globally fitted maximum response, whereas if only one ligand was a full agonist, statistical comparison was performed prior to normalisation, but the numerical results are presented after normalisation to the full agonist response. See Supplementary Fig. 1 for further analysis of β-arrestin-2 recruitment using a different system. ∗p < 0.05 indicated by the statistical test.

GLP-1R						
Mini-Gs				β-arrestin-2		
	pEC_50_ (M)	E_max_ (% max)	K@[max] (min-1)	pEC_50_ (M)	E_max_ (% max)	K@[max] (min-1)
GLP-1	7.7 ± 0.1	105 ± 2	0.30 ± 0.04	7.3 ± 0.2	100 ± 0	1.25 ± 0.31
GLP-1-AIB2	7.9 ± 0.0	96 ± 2	0.21 ± 0.01∗	7.7 ± 0.1∗	68 ± 4∗	0.82 ± 0.10
GCGR						
Mini-Gs				β-arrestin-2		
	pEC_50_ (M)	E_max_ (% max)	K@[max] (min-1)	pEC_50_ (M)	E_max_ (% max)	K@[max] (min-1)
GCG	6.7 ± 0.0	100 ± 0	0.17 ± 0.03	6.1 ± 0.1	100 ± 0	0.89 ± 0.18
GCG-AIB2	6.7 ± 0.1	54 ± 4∗	0.11 ± 0.01	6.2 ± 0.1	25 ± 2∗	0.29 ± 0.03∗
GCG-H3	6.8 ± 0.1	66 ± 5∗	0.13 ± 0.04	6.3 ± 0.1	54 ± 6∗	0.51 ± 0.06∗
GCG-AIB2H3	7.0 ± 0.1∗	74 ± 5∗	0.13 ± 0.01	6.6 ± 0.1∗	53 ± 2∗	0.56 ± 0.06∗

An alternative method for bias quantification has been proposed [29,46] that is applicable to scenarios when kinetic response data is available. This model-free approach quantifies efficacy, termed kτ, from the initial response rate at a saturating agonist concentration. After logarithmic transformation of kτ, bias can be determined by first normalising to a reference ligand (obtaining Δlog kτ) and then comparing responses between pathways (obtaining ΔΔlog kτ). In our study, mini-Gs responses could be fitted as one-phase exponential association curves, whereas β-arrestin-2 showed a characteristic rapid increase and slower decline presumed to reflect β-arrestin association followed by dissociation from the target receptor and required a bi-exponential equation to define the association and dissociation rate constants [47] (Figure 1D). GLP-1-AIB2 showed subtly slower kinetics at GLP-1R for both pathways than did GLP-1, which did not translate into a significant degree of bias using the ΔΔlog kτ method (Table 2 and Figure 1C). At GCGR, mini-Gs and β-arrestin-2 association kinetics were also slower for GCG-AIB2 than glucagon (Figure 1D and Table 2), with bias assessment from the kinetic data again suggesting a preference for mini-Gs coupling that was negated with the introduction of H3 (that is, less bias with GCG-AIB2H3 than GCG-AIB2; Figure 1C).

Overall, these data indicate that introducing the AIB2 substitution into GLP-1 and glucagon led to a noticeable reduction in efficacy for β-arrestin-2 recruitment, more than mini-Gs recruitment, with glucagon more affected than GLP-1. However, at GCGR, this effect could be mitigated by the presence of H3. The Q/H switch at position 3 thereby provides a method of modulating efficacy while retaining AIB2-induced resistance to DPP4.

### 3.2 GCGR molecular dynamics simulations

**Figure 2:**
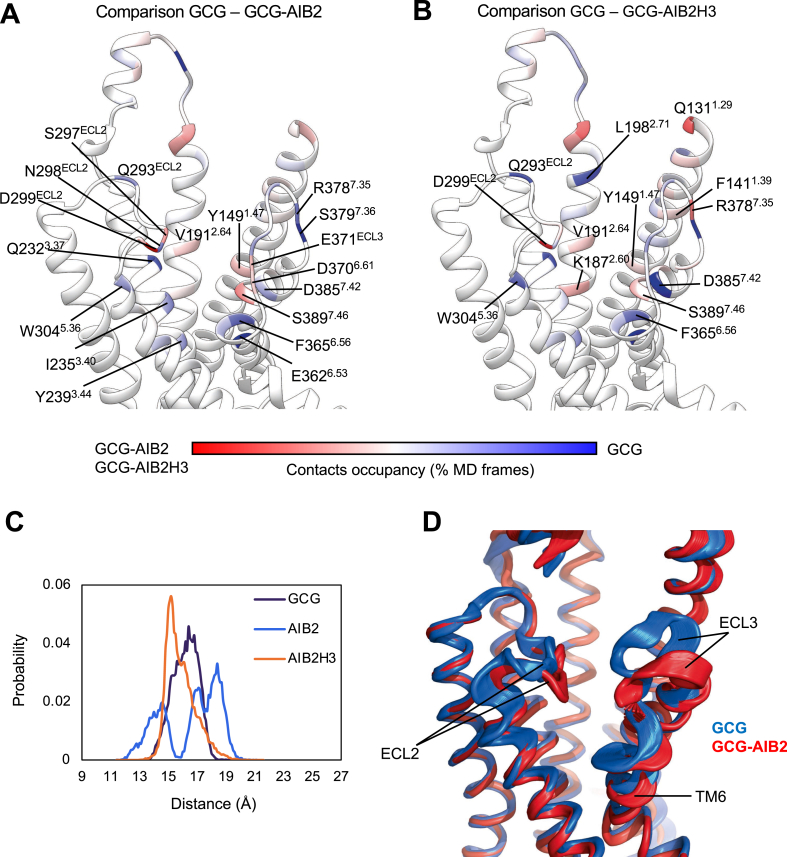
MD simulations of GCGR in complex with glucagon, GCG-AIB2, or GCG-AIB2H3. A and B show the difference in the contacts between GCGR and GCG-AIB2 (A) or GCG-AIB2H3 (B) plotted on the ribbon representation of GCGR; residues in red were more engaged by GCG-AIB2 (A) or GCG-AIB2H3 (B), while blue residues formed more contacts with GCG. (C) Probability distribution of the distance between TM6 residue T369 and the origin of the cartesian coordinates (point 0, 0, 0). (D) Superposition of the PC1 analysis computed on the simulations of GCGR in complex with GCG (blue) or GCG-AIB2 (red).

We performed molecular dynamics simulations of the active state GCGR in complex with glucagon, GCG-AIB2, or GCG-AIB2H3 to retrieve insights into the effects that peptide mutations have on the interactions, fingerprints, and receptor flexibility. Substituting serine at position 2 with the non-standard residue AIB produced a substantial loss of interactions with the top of transmembrane helix 6 (TM6) and TM7 (E3626.53, F3656.56, and D3857.42 in Figure 2A,B). Fewer contacts were also formed with TM3 (I2353.40 and Y2393.44) and TM5 (W3045.36) compared to glucagon. Substituting S2 with the hydrophobic AIB removed a persistent hydrogen bond with a D3857.42 side chain (Table 3) and moved the barycentre of the interactions towards extracellular loop 2 (ECL2) (D299ECL2 and S297ECL2 in Figure 2A,B) due to hydrogen bonds with H1 and T5 (Table 3). The partial release of TM6 from the restraining interactions with the peptide N-termini was corroborated by the high flexibility displayed in Figure 2C. GCGR in complex with glucagon and AIB2H3, on the other hand, was characterised by low plasticity of TM6 as indicated by monodisperse probability curves. Overall, glucagon and GCG-AIB2 stabilised divergent GCGR conformations of TM6, ECL2, and ECL3 (Figure 2D). Interestingly, in the closely related GLP-1R, ECL2 is essential for transducing peptide-receptor interactions into cAMP accumulation, while a possible correlation between peptides more prone to interact with ECL3 and β-arrestin-influenced signalling events such as ERK1/2 phosphorylation has been proposed, for example, for oxyntomodulin, exendin-4 and P5 [38,48]. Significantly, recently described structures of GLP-1R complexed with semaglutide or taspoglutide that contain the AIB2 substitution also highlight divergence in the conformation of ECL3 compared to GLP-1 [49]. Moreover, a recent GCGR structural study identified distinct ECL3 conformations stabilised by glucagon and P15, a GLP-1R/GCGR co-agonist peptide [50].

**Table 3:** Molecular dynamics simulation results. Hydrogen bonds between GCGR and the first five amino acids in GCG, GCG-AIB2, and GCG-AIB2H3. Occupancy represents the number of frames with interactions divided by the total number of frames. ss indicates side chain-side chain hydrogen bonds, while sb indicates backbone-side chain hydrogen bonds.

Peptide	Ligand residue	Receptor residue	Occupancy (%)	Type of hydrogen bond
GCG	H1	E362^6.53^	37.4	sb
		D385^7.42^	30.4	
	S2	D385^7.42^	78.5	ss
			18.4	sb
	Q3	Y145^1.43^	20.5	ss
		Y149^1.47^	18.2	
	T5	N298^ECL2^	10.6	sb
GCG-AIB2	H1	D299^ECL2^	30.2	sb
		K187^2.60^	14.2	ss
		D385^7.42^	11.7	
	AIB2	D385^7.42^	43.2	sb
	Q3	Y149^1.47^	35.1	ss
		S389^7.46^	13.6	
	G4	T296^ECL2^	16.4	sb
	T5	D299^ECL2^	30.4	ss
GCG-AIB2H3	H1	D299^ECL2^	47.2	sb
		K187^2.60^	2.46	ss
		N298^ECL2^	14.1	
	AIB2	D385^7.42^	21.0	sb
	H3	Y149^1.47^	11.9	ss
	T5	D299^ECL2^	13.8	

The simulations suggested that the Q3H mutation introduced in GCG-AIB2H3 favoured interactions between the peptide and TM2 residues K1872.60, V1912.64, and Q1311.29 located on the receptor's stalk region. K1872.60 in particular is part of the conserved hydrophilic region within class B receptor TMD implied in binding, functionality, and signal transmission [51]. It is plausible that the recovery in efficacy displayed by GCG-AIB2H3 over AIB2 might be driven by stronger interactions with TM2. Moreover, the whole TMD closed up around GCG-AIB2H3 during the simulations, similar to GCG (Figure 2C,D).

### 3.3 Pharmacologically stabilised GLP-1R/GCGR co-agonists to study the impact of efficacy variations

A pair of peptides termed SRB103 (Table 1) was developed by an iterative process of sequence changes to the GLP-1R/GCGR co-agonist used in an earlier study [25]. As the previous peptide was derived from OXM, it contained the N-terminal sequence H–S-Q, which was modified to H-AIB-Q (SRB103Q) or H-AIB-H (SRB103H) along with additional conservative changes to enhance physicochemical properties such as stability and solubility. As expected, both SRB103Q and SRB103H were highly resistant to DPP-4-mediated degradation (Supplementary Fig. 2A).

**Figure 3:**
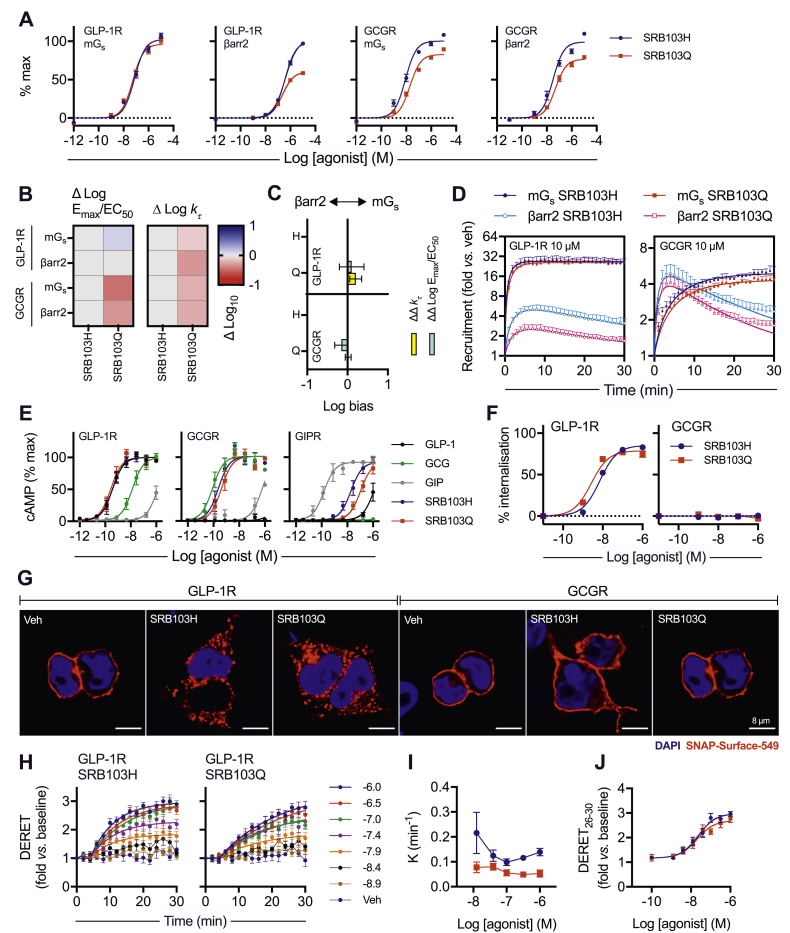
Development of a DPP-4-resistant GLP-1R/GCGR co-agonist with variable efficacy for intracellular effectors. (A) Concentration responses with 3-parameter fits for SRB103H- and SRB103Q-induced recruitment of mini-Gs or β-arrestin-2 to GLP-1R-SmBiT or GCGR-SmBiT in HEK293T cells, n = 6. (B) Heatmap representation of mean responses after quantification by log(max/EC50) or kτ method and normalisation to SRB103H as the reference ligand. (C) Assessment of bias between mini-Gs and β-arrestin-2 recruitment from log(max/EC50) or the kτ method. The 95% confidence intervals are shown to allow identification of ligands with statistically significant bias vs the reference ligand SRB103H. (D) Single maximal concentration kinetic responses for each ligand/receptor/pathway combination using data shown in (A), with one-phase association fits for mini-Gs and bi-exponential fits for β-arrestin-2. (E) cAMP responses in PathHunter CHO–K1 cells stably expressing GLP-1R, GCGR, or GIPR, n = 6, with 3-parameter fits shown. (F) SNAP-GLP-1R and SNAP-GCGR internalisation measured by high content analysis (HCA) in HEK293 cells, n = 4, with 3-parameter fits shown. (G) Representative images from n = 2 experiments showing endocytosis of SNAP-tagged receptors transiently expressed in HEK293 cells and treated with 100 nM of agonist for 30 min. Scale bars = 8 μm. (H) SNAP-GLP-1R internalisation in HEK293-SNAP-GLP-1R cells, n = 5, with one-phase association fits for ligand concentrations > 10 nM shown (expressed as log[agonist] in M). (I) The concentration dependency of internalisation kinetics from (H) is shown. (J) Concentration responses quantified from the average response during the last 3 time points from (H), with 3-parameter fits. Data are represented as mean ± SEM for concentration response curves or 95% confidence intervals for bias plots; bias data are considered significant if the 95% confidence interval does not cross 0.

The mini-Gs and β-arrestin-2 recruitment profiles of each ligand were compared at both GLP-1R and GCGR (Figure 3A, Table 4, and Supplementary Fig. 2B). At GLP-1R, AUC analysis from the kinetic response data indicated a 40% reduction in β-arrestin-2 efficacy but a small increase in potency for the AIB2Q3 ligand compared to AIB2H3, with the mini-Gs response unaffected. At GCGR, both potency and efficacy were significantly reduced in both pathways with the AIB2Q3 ligand, although the magnitude of the efficacy reduction (∼20%) was small compared to the same sequence substitutions when applied to glucagon in Figure 1. Using the log(max/EC50) method, there was no statistically significant bias between mini-Gs and β-arrestin-2 for SRB103Q vs SRB103H at either receptor (Figure 3B,C). However, bias estimates from the kinetic responses (ΔΔlog kτ method) suggested a subtle preference for SRB103Q at GLP-1R towards mini-Gs recruitment (Figure 3C,D). As a role for Gαq signalling has been reported for GLP-1R in islets [52], and Gαi-dependent signalling was shown to paradoxically increase GCGR-induced hepatic glucose output [53], we also compared each SRB103 ligand for its ability to promote mini-Gq and mini-Gi to GLP-1R and GCGR (Supplementary Figs. 2C and D). These responses were generally of considerably lower magnitude than for mini-Gs, suggesting that Gαs dominates in this cell system. However, SRB103H appeared to induce greater coupling to mini-Gq and mini-Gi than SRB103Q to GCGR.

**Table 4:** Pharmacological evaluation of SRB103H3 vs SRB103. Mean ± SEM parameter estimates from 3-parameter fitting of data from Figure 3, Figure 4 and association rate constants for kinetic data where relevant. Statistical comparisons performed by paired t-tests comparing SRB103Q vs SRB103H. If both ligands were full agonists, Emax values are shown after re-fitting data normalised to the globally fitted maximum response. If only one ligand was a full agonist, statistical comparison was performed prior to normalisation, but the numerical results are presented after normalisation to the full agonist response. ∗p < 0.05 indicated by the statistical test.

	SRB103H			SRB103Q		
	pEC_50_ (M)	E_max_	K@[max] (min-1)	pEC_50_ (M)	E_max_	K@[max] (min-1)
GLP-1R mini-G_s_ (HEK293T)	7.1 ± 0.1	103 ± 4	0.93 ± 0.29	7.3 ± 0.1	97 ± 3	0.70 ± 0.20∗
GLP-1R βarr2 (HEK293T)	6.4 ± 0.1	100	0.37 ± 0.06	6.7 ± 0.0∗	60 ± 2∗	0.39 ± 0.05
GCGR mini-G_s_ (HEK293T)	8.1 ± 0.1	100	0.14 ± 0.01	7.7 ± 0.1∗	83 ± 1∗	0.13 ± 0.01
GCGR βarr2 (HEK293T)	7.5 ± 0.1	100	0.81 ± 0.07	7.3 ± 0.1∗	77 ± 2∗	0.71 ± 0.06∗
GLP-1R cAMP (CHO–K1)	9.5 ± 0.1	99 ± 4	n.c.	9.5 ± 0.1	103 ± 2	n.c.
GCGR CAMP (CHO–K1)	9.6 ± 0.2	107 ± 5	n.c.	9.4 ± 0.2	103 ± 4	n.c.
GIPR CAMP (CHO–K1)	7.6 ± 0.2	101 ± 5	n.c.	7.0 ± 0.1∗	92 ± 9	n.c.
DERET (HEK293-SNAP-GLP-1R)	7.7 ± 0.2	3.0 ± 0.1	0.14 ± 0.02	7.7 ± 0.2	2.7 ± 0.2∗	0.5 ± 0.01∗
HCA assay (HEK293-SNAP-GLP-1R)	8.1 ± 0.1	86 ± 2		8.6 ± 0.0∗	79 ± 3∗	
INS-1 832/3 cAMP, 10 min	8.1 ± 0.1	67 ± 6		8.2 ± 0.2	70 ± 7	
Primary islet cells cAMP, 5 min	9.0 ± 0.4	1.7 ± 0.1		9.0 ± 0.4	1.6 ± 0.1	
INS-1 832/3 insulin secretion, 16 h	8.8 ± 0.2	3.5 ± 0.3		9.3 ± 0.1∗	3.4 ± 0.4	
Huh7-GCGR cAMP, 10 min	10.5 ± 0.2	27 ± 4		10.8 ± 0.2∗	27 ± 4	
Huh7-GCGR cAMP,16 h	9.6 ± 0.2	173 ± 11		9.7 ± 0.2	284 ± 38∗	
cAMP 10 min (primary hepatocytes)	9.4 ± 0.1	170 ± 7		8.9 ± 0.1∗	175 ± 12	
cAMP 16 h (primary hepatocytes)	7.6 ± 0.1	206 ± 7		7.5 ± 0.0	200 ± 6	

The cAMP signalling responses were also assessed in CHO–K1 cell lines expressing GLP-1R, GCGR, or glucose-dependent insulinotropic polypeptide receptor (GIPR) (Figure 3E and Table 4). Unsurprisingly given the high degree of amplification seen in heterologous cell lines, reduced mini-Gs recruitment efficacy did not result in any reduction in cAMP Emax with SRB103Q, similar to a recent evaluation of GLP-1R/GIPR co-agonists [54]. Potencies for SRB103Q and SRB103H were, as expected, indistinguishable at GLP-1R, with a non-significant reduction for SRB103Q at GCGR. Both ligands showed at least 100-fold reduced potency for GIPR cAMP signalling compared to GIP itself, even in this highly amplified heterologous system, suggesting that GIPR was unlikely to contribute to their overall metabolic actions.

A close correlation was previously observed between transducer coupling efficacy and ligand-induced endocytosis of GLP-1R [19,20]. GCGR, on the other hand, appears to internalise far more slowly [33,55]. We investigated the effects of SRB103Q and SRB103H on internalisation of GLP-1R and GCGR SNAP-tagged at their N-termini in HEK293T cells using high content imaging [33]. Both ligands induced pronounced GLP-1R internalisation, with a minor reduction in efficacy with SRB103Q, but GCGR barely internalised with either ligand (Figure 3F); higher resolution images of SNAP-GLP-1R- or SNAP-GCGR-expressing cells labelled prior to agonist treatment corroborated these findings (Figure 3G). Interestingly, when measured by diffusion-enhanced resonance energy transfer (DERET) [56], kinetics of GLP-1R internalisation were considerably slower for SRB103Q than SRB103H throughout the concentration range (Figure 3H,I), although using AUC quantified from the end of the stimulation period, SRB103Q internalisation efficacy was only subtly reduced (Figure 3J), similar to the result in the high content imaging assay.

These data indicate that the AIB2Q3 iteration of SRB103 showed reduced efficacy for recruitment of β-arrestin-2 at GLP-1R and, to a lesser degree, for mini-Gs and β-arrestin-2 at GCGR.

### 3.4 Evaluating acute vs prolonged responses with SRB103H and SRB103Q

**Figure 4:**
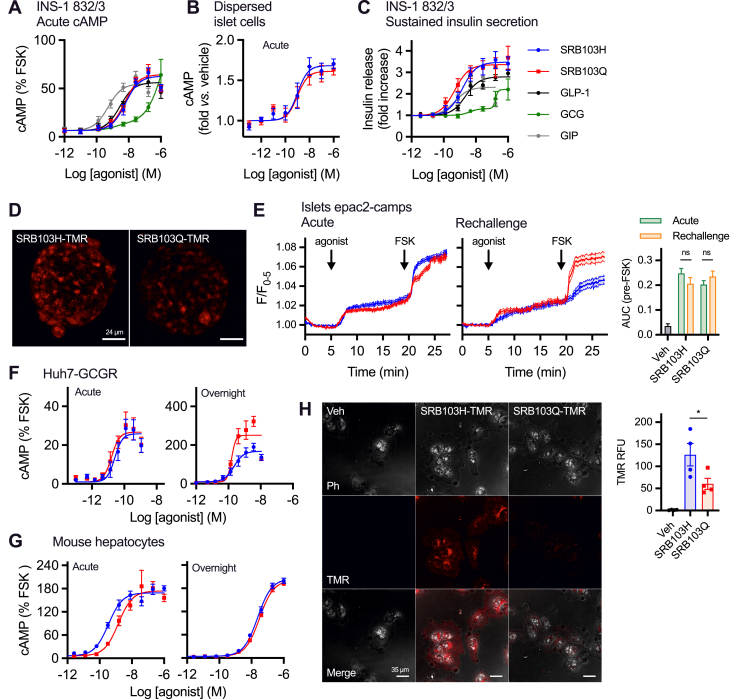
Acute vs prolonged responses in vitro with SRB103Q and SRB103H. (A) Acute cAMP signalling in INS-1 832/3 cells, 10 min stimulation with 100 μM of IBMX, n = 5, 3-parameter fits shown (biphasic fit for GCG). (B) Acute cAMP signalling in primary dispersed mouse islets, 5 min stimulation with 500 μM of IBMX, n = 4, 3-parameter fits shown. (C) Cumulative insulin secretion from overnight stimulation of INS-1 832/3 cells, n = 5, 3-parameter fits shown (biphasic fit for GCG). (D) TMR agonist uptake in intact mouse islets, representative of n = 2 repeats, maximum intensity projections are shown. (E) Whole islet cAMP responses to stimulation with 100 nM of the indicated agonist acutely or after 4-h pre-treatment and washout measured by FRET with virally transduced epac2-camps. Quantification from 25 to 42 mouse islets per treatment (5–9 mice from at least 2 independent islet preparations). AUCs during the agonist exposure period (pre-forskolin [10 μM]) were quantified and compared by two-way ANOVA with Sidak's test. Representative images are shown. (F) Acute (10 min) and sustained (16 h) cAMP accumulation in Huh7-GCGR cells expressed relative to 10 μM of forskolin response, n = 6. (G) Acute (10 min) and sustained (16 h) cAMP accumulation in primary mouse hepatocytes, expressed relative to 10 μM of forskolin response, n = 4. (H) TMR-ligand uptake in Huh7-GCGR cells, 30 min stimulation at 100 nM, representative images from n = 4 repeats with quantification as relative fluorescence units (RFU) and comparison by paired t-test. Data are represented as mean ± SEM, with individual repeats in some cases.

Reductions in efficacy for β-arrestin-2 recruitment and endocytosis lead to prolongation of cAMP signalling at GLP-1R [19,41] and GCGR [33], which is thought to result from avoidance of target receptor desensitisation and/or downregulation. Interestingly, despite the canonical role of β-arrestins in promoting clathrin-mediated endocytosis, β-arrestin recruitment appears to be dispensable for GLP-1R internalisation [23,33], indicating that these phenomena may modulate signalling duration through distinct mechanisms. In both INS-1 832/3 clonal beta cells [24] and dispersed mouse islet cells, biochemically measured acute cAMP responses to SRB103Q and SRB103H were indistinguishable (Figure 4A, B, and Table 4). However, SRB103Q showed greater potency than SRB103H for prolonged insulin secretion in INS-1 832/3 cells, amounting to, for example, an almost two-fold increase in insulin release at ∼1 nM agonist concentration (Figure 4C and Table 4). Of note, uptake in mouse islets of an SRB103Q analogue conjugated to tetramethylrhodamine (TMR) close to the C-terminus (SRB103Q-TMR, Table 1) was somewhat reduced compared to SRB103H-TMR (Figure 4D) in keeping with the moderate differences in GLP-1R endocytosis observed with this ligand in Figure 3. Additionally, FRET imaging of intact mouse islets virally transduced to express the cAMP sensor epac2-camps [57] demonstrated that both agonists acutely induced similar cAMP dynamics (Figure 4E), but when pre-treated for 4 h with each ligand and then rechallenged after a washout period, a trend towards reduced responsiveness for SRB103H was observed. This difference was not significant when quantified from the whole re-stimulation period, but it was clearly observed that the epac2-camps average signal increase on SRB103H rechallenge was slower than for SRB103Q (k = 0.28 vs 0.53 min-1 from pooled responses to SRB103H and SRB103Q, respectively), suggesting diminished responsiveness with the former ligand.

We also assessed the potential for time-dependent differences in GCGR signalling. For islet cells, maximal acute cAMP responses in Huh7 hepatoma cells stably expressing GCGR [25] were indistinguishable, but a clear increase was seen with SRB103Q when the cells were incubated for 16 h (Figure 4F and Table 4). GCGR responses were also evaluated in primary mouse hepatocytes; SRB103Q showed reduced potency acutely, but after overnight treatment, this difference disappeared (Figure 4G and Table 4). Interestingly, although SNAP-GCGR endocytosis was barely detectable in HEK cells (see Figure 3), TMR-conjugated SRB103H and SRB103Q analogues were clearly taken up into punctate endosome-like structures in Huh7-GCGR cells, with greater uptake seen with the H3 ligand (Figure 4H). This apparent discrepancy between receptor and ligand internalisation might be explained by rapid dissociation of the endocytosed GCGR/agonist complex and subsequent recycling of the receptor to the plasma membrane.

Overall, these studies indicate a general tendency for SRB103Q responses at both receptors to be relatively enhanced with longer stimulations, which is compatible with reduced β-arrestin-mediated desensitisation of this ligand compared to SRB103H.

### 3.5 Anti-hyperglycaemic responses were prolonged after a single dose of SRB103Q vs SRB103H in mice

**Figure 5:**
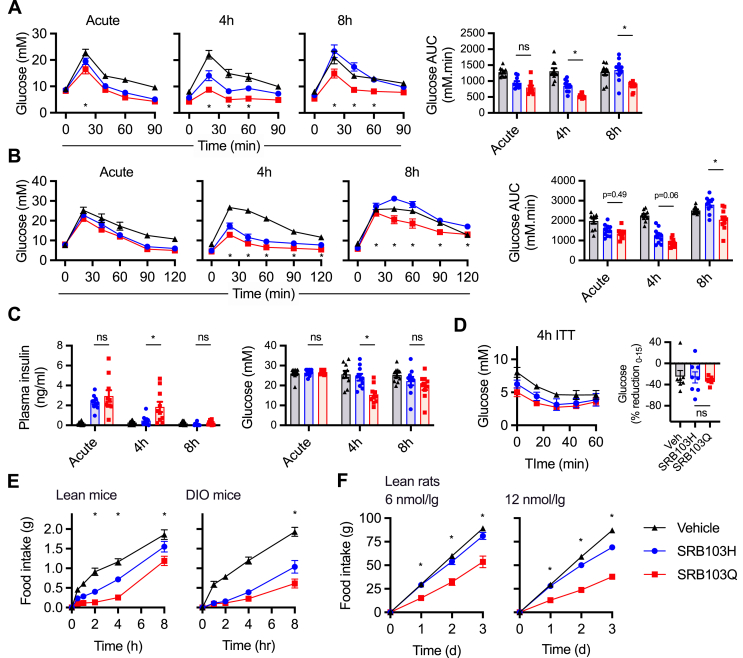
Immediate and delayed responses to SRB103Q and SRB103H in mice. (A) Blood glucose results during intraperitoneal glucose tolerance tests (IPGTTs) performed in lean male C57Bl/6 mice (n = 10/group) with 2 g/kg of glucose injected IP at the same time as, 4 h after, or 8 h after 10 nmol/kg of agonist injection. Time point and AUC comparisons both by repeated measures two-way ANOVA with Tukey's test; only SRB103Q vs SRB103H comparisons are shown. (B) As in (A) but in diet-induced obese male C57Bl/6 mice. (C) Plasma insulin and blood glucose results in lean male C57Bl/6 mice (n = 10/group) 10 min after 2 g/kg of IP glucose administration, concurrently with, 4 h after, or 8 h after 10 nmol/kg of agonist injection. AUC comparisons by repeated measures two-way ANOVA with Tukey's test; only SRB103Q vs SRB103H comparisons are shown. (D) Blood glucose during insulin tolerance test (0.75 U/kg of recombinant human insulin IP) performed 4 h after administration of 10 nmol/kg of agonist injection in lean male C57Bl/6 mice (n = 8/group). Percentage reduction from 0 to 15 min is shown and compared by one-way ANOVA with Tukey's test; only SRB103Q vs SRB103H comparison is shown. (E) Food intake in overnight-fasted lean male C57Bl/6 mice (n = 8/group) treated with 10 nmol/kg of indicated agonist. Time point comparisons both by repeated measures two-way ANOVA with Tukey's test; only SRB103Q vs SRB103H comparisons are shown. (F) Food intake in overnight-fasted lean Sprague Dawley rats (n = 6–7/group) treated with indicated agonist dose every 24 h. Time point comparisons both by repeated measures two-way ANOVA with Tukey's test; only SRB103Q vs SRB103H comparisons are shown. ∗p < 0.05 indicated by the statistical test. Data are represented as mean ± SEM with individual replicates where possible.

As GLP-1R agonists with reduced β-arrestin-2 recruitment efficacy and/or delayed endocytosis show progressive increases in anti-hyperglycaemic efficacy over longer exposure periods [19,21,58], we aimed to establish if this therapeutic principle could also be applied to GLP-1R/GCGR co-agonism. Indeed, blood glucose concentrations during an intraperitoneal glucose tolerance test (IPGTT) in lean mice tended to be lower after a single administration of SRB103Q compared to SRB103H, with this difference enhanced by a longer agonist exposure time (Figure 5A). A similar pattern was seen at a range of agonist doses (Supplementary Fig. 3A) and in diet-induced obese (DIO) mice (Figure 5B).

Both GLP-1R and GCGR agonism potentiate glucose-stimulated insulin secretion [12], but can also acutely enhance insulin-stimulated glucose disposal [11,59]. Plasma insulin concentrations measured 10 min into a 4-h-delayed IPGTT were higher with SRB103Q than SRB103H treatment, suggesting the former's improved anti-hyperglycaemic efficacy is likely to derive at least partly from action on beta cells (Figure 5C) in keeping with greater insulin release observed after prolonged stimulation with SRB103Q in Figure 4. We also performed insulin tolerance tests (ITTs) 4 h after agonist administration to assess potential effects on insulin sensitivity (Figure 5D and Supplementary Fig. 3B). While ITT interpretation was complicated by differences in baseline due to the prior agonist exposure period, neither absolute nor percentage reductions in blood glucose levels were different between agonists. Appetite suppression was also assessed in lean and diet-induced obese mice. SRB103Q was more effective than SRB103H, particularly at later time points in the obese cohort (Figure 5E). Additional studies in lean rats confirmed that the anorectic effect of SRB103Q was greater than SRB103H over 72 h (Figure 5F). Plasma concentrations of each ligand were the same 4 h after a single dose in mice (Supplementary Fig. 3C), suggesting that the progressive divergence in physiological effects several hours after dosing was unlikely to be due to altered pharmacokinetics. Serial sampling in rats with peptide co-injected subcutaneously with zinc to slow absorption through depot formation also indicated no obvious difference in pharmacokinetics (Supplementary Fig. 3D).

Overall, these results indicate that, despite showing lower acute efficacy for intracellular effector recruitment at both GLP-1R and GCGR, SRB103Q showed greater bioactivity in mice than SRB103H. For glycaemic effects, this difference tended to become more apparent with time in keeping with the previously established principle that the metabolic advantages of biased GLP-1R agonists are temporally specific.

### 3.6 Improved anti-hyperglycaemic efficacy of SRB103Q was preserved with chronic administration

**Figure 6:**
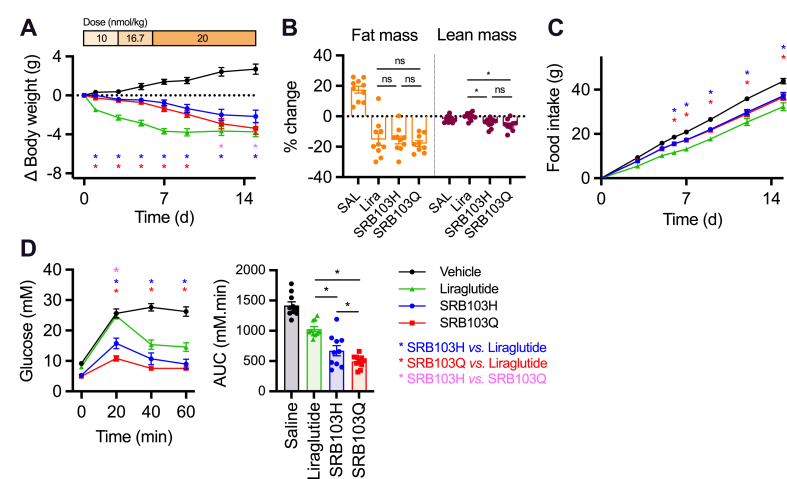
Repeated administration of SRB103Q and SRB103H. (A) Effect on body weight of daily administration by s.c. injection of SRB103Q, SRB103H, liraglutide, or vehicle on body weight in male diet-induced obese C57Bl/6 mice, n = 10/group, with statistical comparisons between agonists by repeated measures two-way ANOVA with the Holm–Sidak test. The injected daily dose is indicated above the graph. (B) Body composition analysis by EchoMRI performed at the end of the study, with changes from baseline compared between treatments by one-way ANOVA with the Holm–Sidak test. (C) As in (A) but cumulative food intake. (D) IPGTT (2 g/kg of glucose) performed on day 15 of the study 8 h after agonist administration. Statistical comparisons by repeated measures two-way ANOVA with the Holm–Sidak test (time points) or one-way ANOVA with the Holm–Sidak test. ∗p < 0.05 indicated by the statistical test and colour-coded where applicable. Data are represented as mean ± SEM with individual replicates where possible.

GLP-1R/GCGR co-agonists may hold advantages over GLP-1R mono-agonists for treating obesity and related metabolic diseases as their GCGR-mediated effects on energy expenditure can promote additional weight loss [35,60,61]. To determine if the apparent benefits of SRB103Q on glucose homeostasis revealed in single-dose studies are also maintained after repeated dosing, SRB103H, SRB103Q, and the GLP-1R mono-agonist liraglutide were administered at matched doses to DIO mice for 2 weeks. The dose was up-titrated over several days, analogous to typical practise in the clinic, as well as in preclinical studies of incretin receptor agonists [21,62]. As expected, all of the agonists led to a significant amount of weight loss compared to vehicle (Figure 6A). This was primarily due to fat mass loss, although interestingly, a small amount of lean mass was lost with both SRB103 peptides but not liraglutide (Figure 6B), which could result from the known effects of GCGR agonism on amino acid flux and muscle catabolism [63]. However, the trajectory for weight lowering differed for both dual GLP-1R/GCGR agonists compared to liraglutide, with the latter being more effective earlier in the study before reaching a plateau after one week, as commonly observed with GLP-1R mono-agonists in rodents [[64], [65], [66]]. Importantly, weight loss with both dual agonists was achieved despite liraglutide being more effective at suppressing energy intake throughout the study (Figure 6C), suggesting a contribution of increased energy expenditure [67]. Interestingly, SRB103Q was moderately more effective for weight loss than SRB103H despite similar energy intake, raising the possibility that reduced GCGR desensitisation could have contributed to improved longer-term effects on energy expenditure. Both SRB103Q and SRB103H outperformed liraglutide in an IPGTT performed at the end of the study, with SRB103Q being the most effective at reducing the glucose excursion (Figure 6D).

## 4 Discussion

In this study, we carefully evaluated the effects on GLP-1R and GCGR activity of the AIB2 substitution commonly used to confer DPP-4 resistance to therapeutic GLP-1R/GCGR peptide agonists. Depending on the peptide context, this substitution reduced efficacy for recruitment of key intracellular effectors at both target receptors. Interestingly, this effect was counteracted by substituting the neighbouring amino acid Q to H, providing a method to compare the impact of the resultant efficacy changes while retaining DPP-4 resistance. While the efficacy-reducing effect of AIB2 was most prominently observed with glucagon at GCGR, in the context of the SRB103 peptides, this effect was in fact greater at GLP-1R, specifically for β-arrestin-2 recruitment, although GCGR responses were also modestly reduced. The potential importance of this pharmacological finding was hinted at by studies in primary and clonal cell models of pancreatic beta cells (or islets) and liver, tissues in which these responses are chiefly driven by, respectively, GLP-1R and GCGR, where we observed that the lower efficacy SRB103Q ligand showed at least a trend towards relatively enhanced signalling responses at both GLP-1R and GCGR over time. These observations support our in vivo findings that the improved anti-hyperglycaemic performance of SRB103Q becomes more apparent at later time points after dosing, as was previously seen with GLP-1R mono-agonists with analogous signalling parameters [19].

This study was originally designed to assess the potential for biased agonism to improve therapeutic targeting of GLP-1R and GCGR. However, the magnitude of bias between SRB103Q and SRB103H as assessed by two validated models was relatively small. Interestingly, while biased agonism has recently attracted considerable attention, it has also been suggested that low intrinsic signalling efficacy, rather than biased agonism per se, is a viable alternative explanation for the improved performance of certain μ opioid receptor agonists [68], a GPCR target usually considered highly tractable to biased agonism [69]. This possibility is reinforced by a lack of consistency between formal bias estimates obtained from different analytical approaches, which can lead to different conclusions from the same data [70]. With regard to the lower efficacy SRB103Q agonist in our study, signal amplification downstream of Gαs activation means full cAMP/PKA responses are still possible, so, in combination with reduced efficacy for β-arrestin recruitment, this could lead to reductions in desensitisation over time and allow longer-lasting signalling responses. Thus, beneficial responses from partial agonism may be achieved irrespective of whether or not formally quantified bias is present. Further evaluations to establish whether partial agonism or bias is the most important factor will be required to settle this issue.

AIB2 substitution at position 2 is one of a number of sequence modifications that have been trialled to obtain DPP-4 resistance for incretin receptor analogues. While exendin-4, the prototypical DPP-4 resistant GLP-1R mono-agonist, contains a glycine at position 2, GLP-1-G2 was recognised in early studies to show an unacceptable loss of signalling potency [71]; more recently, it was demonstrated that this is also associated with reduced efficacy for recruitment of both mini-Gs and β-arrestin-2 to GLP-1R [41]. AIB2 is better tolerated by GLP-1 than G2 while retaining identical protection against DPP-4-mediated degradation [71] and has been incorporated into the current leading GLP-1R mono-agonist semaglutide [72]. Our new data indicate that, in the context of native GLP-1, AIB2 leads to a significant reduction in efficacy for recruitment of β-arrestin-2 while barely affecting recruitment of mini-Gs. This effect is likely to be peptide-specific, as we did not observe similar reductions in β-arrestin recruitment by AIB2 containing semaglutide in a previous study [19]. Interestingly, in the present work, AIB2 led to marked attenuation of engagement of GCGR with intracellular effectors by glucagon analogues, an effect that was previously hinted by the lower cAMP signalling potency with a glucagon analogue bearing AIB at positions 2 and 16 [73]. In the latter study, GCGR signalling was partly restored by conjugation to a fatty acid moiety, a well-established strategy used primarily to extend peptide pharmacokinetics by promoting reversible binding to albumin but, in this case, also found to enhance receptor activation. In our study, we observed that switching Q to H at position 3 of glucagon was an alternative method of reversing the deleterious effect of AIB2 on GCGR signalling. It is not clear if these strategies are equivalent, as in Ward et al.‘s study [73], the signalling deficit seen with AIB2 was a reduction in cAMP potency, whereas in our study, efficacies for mini-Gs and β-arrestin-2 reduced but potencies were unaffected. A recent evaluation of GLP-1R/GCGR co-agonists [22] showed that the GLP-1R/GCGR/GIPR “tri-agonist” (GLP-1R/GCGR/GIPR) peptide originally described by Finan et al. [74], which includes the N-terminal sequence H-AIB-Q, does indeed show reductions in β-arrestin recruitment efficacy to GLP-1R (modest) and GCGR (substantial) compared to the endogenous agonist without major loss in potency, broadly matching our observations with native ligand analogues and SRB103 peptides. Measured signalling potency, especially in the context of significantly amplified responses, for example, cAMP, is driven to varying extents by both affinity and efficacy, with our results highlighting how the standard approach to evaluating incretin receptor agonists in vitro using cAMP in heterologous systems, which tends to render all compounds full agonists, may be insufficient to adequately decipher ligand pharmacology [75]. Importantly, our study also provides structural insights into the importance of position 2 of glucagon peptide analogues, with molecular dynamics simulations indicating that the reduced β-arrestin-2 recruitment associated with the AIB2 substitution was related to reduced engagement of ECL3, a region recently noted to be important for signalling divergence between AIB2-containing GLP-1R agonists including semaglutide or taspoglutide compared to GLP-1 itself [49].

The most striking results in our study were observed from in vivo comparisons of SRB103Q and SRB103H. Here, the lower efficacy SRB103Q (at both GLP-1R and GCGR) peptide outperformed SRB103H for its ability to lower blood glucose levels 4 and 8 h after a single injection despite apparently equivalent pharmacokinetics. While a formal pharmacokinetic study would be required to rule out subtle differences, the a priori expectation that the two ligands would show altered pharmacokinetics is low. These findings are reminiscent of observations with exendin-phe1, a GLP-1R mono-agonist with marked reductions in β-arrestin recruitment efficacy, which displayed better anti-hyperglycaemic effects and increased insulin secretion compared to exendin-4 in mice, with these differences being most obvious at later time points [19]. However, one of the challenges with our study was identifying whether the observed effects resulted from enhanced action primarily at GLP-1R or GCGR, as SRB103Q displayed reduced β-arrestin-2 recruitment efficacy at both receptors, meaning that longer-lasting signalling through avoidance of target desensitisation could apply in both cases. Receptor and/or ligand uptake studies also suggested that endocytosis of both receptors was slower with SRB103Q. Overall, we favour a primarily GLP-1R-mediated mechanism for the observed physiological effects because 1) the selective reduction in β-arrestin-2 recruitment with SRB103Q was larger at GLP-1R than at GCGR and 2) the effect was associated with increases in insulin release and supported by a trend towards reduced islet desensitisation in vitro. While glucagon can augment glucose-stimulated insulin secretion, this effect is mediated mainly by cross-reactivity at GLP-1R [12]. We consider it unlikely that the lower blood glucose levels with SRB103Q resulted from decreased hepatic glucose output via the subtly reduced efficacy of this peptide at GCGR, as it retained full cAMP activity in mouse hepatocytes (and in fact showed progressively greater GCGR-mediated cAMP responses in the Huh7-GCGR model after prolonged stimulation), and the observed glycaemic effects were related mainly to the ability to restrain the hyperglycaemic effect of exogenously administered glucose. For similar reasons, it is improbable that reduced GCGR coupling to Gαi with SRB103Q is the primary reason for its advantageous glycaemic effects, although this remains an outside possibility given the recent observation that GCGR-mediated hepatic glucose output is paradoxically increased by Gαi-dependent JNK activation [53]. Nevertheless, further studies into possible effects of biased or partial agonism at GCGR are warranted and could include studies of lipid metabolism, amino acids, energy expenditure, and more. As antagonists for GLP-1R and GCGR are generally unable to cleanly and completely inhibit the action of high-affinity exogenously administered agonists at pharmacological doses, studies of GLP-1R and GCGR knockout mice will be needed to distinguish each receptor's relative contributions. The well-known phenotype of GCGR knockout mice, which are highly resistant to hyperglycaemia and show other metabolic abnormalities [76], may introduce additional challenges.

SRB103Q and SRB103H were compared in a chronic administration study with liraglutide also included for reference as an exemplar GLP-1R mono-agonist. The important observation was that the enhanced anti-hyperglycaemic benefits of SRB103Q were retained after 2 weeks of repeated administration, suggesting that the apparent benefits of its intracellular signalling profile on glucose homeostasis did not diminish with time. To the best of our knowledge, this represents the first demonstration of the possibility of achieving more effective metabolic control through partial agonism in the context of a GLP-1R/GCGR co-agonist. Notably, somewhat greater weight loss without a corresponding reduction in food intake for SRB103Q was observed, which could conceivably have resulted from increases in sustained GCGR activation as might be predicted from the reduced β-arrestin-2 recruitment efficacy of this peptide compared to SRB103H. The glycaemic effects of both molecules compared favourably with liraglutide at the same dose, although differences in the pharmacokinetics (longer with liraglutide) and amount of bioactive free peptide (lower with liraglutide due to albumin binding) complicated interpretation. Nevertheless, the observation that both SRB103 peptides achieved similar weight loss to liraglutide despite a less potent anorectic effect adds to the evidence that GLP-1R/GCGR co-agonism may be an effective method of treating obesity, potentially with reduced anorexia-associated nausea (although this was not tested directly in our study).

Darbalaei et al.‘s recent study provided a comprehensive description of the pharmacology of other published GLP-1R/GCGR co-agonists [22], including two ligands for which clinical data are available: cotadutide (MEDI0382) [8] and SAR425899 [77]. Neither of these clinical candidate molecules include AIB2 at position 2, but both showed reduced recruitment of β-arrestin-2 to GLP-1R, albeit the reduction was not as great as for SRB103Q. Both also showed significantly reduced recruitment of β-arrestin-2 to GCGR compared to glucagon, a difference that was larger than GCGR efficacy reduction seen with SRB103Q compared to SRB103H. Thus, cotadutide and SAR425899 may well be additional examples of incretin receptor ligands retrospectively identified as showing biased agonist properties as was recently found for the dual GLP-1R/GIPR agonist tirzepatide [54,78,79]. However, the recorded cAMP potencies for cotadutide and SAR425899 in Darbalaei et al.‘s study relative to the endogenous comparator ligands were orders of magnitude less than previously reported [77,80], raising the possibility that the cellular systems used to evaluate these ligands' pharmacology could have affected the results.

In conclusion, our study should be seen as an evaluation of the potential for reduced efficacy to be incorporated into the assessment process for candidate dual GLP-1R/GCGR agonists. Further molecular optimisations, for example, acylation for extended pharmacokinetics, will be required to generate viable molecules for eventual clinical use. Molecular dynamics simulations indicated the relevant differences in engagement with ECL2 and ECL3 that can be used to guide these optimisations. Detailed mechanistic research is also needed to establish the relative contributions of G protein- and β-arrestin-mediated effects at both GLP-1R and GCGR and will help clarify how investigational incretin receptor agonists are prioritised during drug development for T2D and obesity.

## Funding acknowledgements

The Section of Endocrinology and Investigative Medicine is funded by grants from the 10.13039/501100000265MRC, 10.13039/501100000268BBSRC, and NIHR and is supported by the 10.13039/100014461NIHR Biomedical Research Centre Funding Scheme. The views expressed are those of the authors and not necessarily those of the funders. D.J.H. was supported by 10.13039/501100000265MRC (MR/N00275X/1 and MR/S025618/1) and 10.13039/501100000361Diabetes UK (17/0005681) project grants. This project received funding from the 10.13039/501100000781European Research Council (ERC) under the 10.13039/501100007601European Union's Horizon 2020 Research and Innovation Programme (Starting Grant 715,884 to D.J.H.). A.T. acknowledges funding from 10.13039/501100000361Diabetes UK. B.J. acknowledges support from the 10.13039/501100000691Academy of Medical Sciences, 10.13039/501100000382Society for Endocrinology, 10.13039/100014669British Society for Neuroendocrinology, and an 10.13039/501100000266EPSRC capital award. B.J. and A.T. also received funding from the 10.13039/501100000265MRC (MR/R010676/1) and the European Federation for the Study of Diabetes. E.R.M. acknowledges support from 10.13039/501100000297Royal College of Surgeons of England and 10.13039/501100000265MRC clinical research training fellowships.
