# Supplementary material for: Partial agonism improves the anti-hyperglycaemic efficacy of an oxyntomodulin-derived GLP-1R/GCGR co-agonist
Source: Mol Metab. 2021 Apr 30;51:101242. doi: 10.1016/j.molmet.2021.101242 (PMC8163982; doi:10.1016/j.molmet.2021.101242)
Supplement: Multimedia component 1 [file mmc1.docx]

Supplementary Information

Partial agonism improves the anti-hyperglycaemic efficacy of an oxyntomodulin-derived GLP-1R/GCGR co-agonist

Phil Pickford^1^, Maria Lucey^1^, Roxana-Maria Rujan^2^, Emma Rose McGlone^1^, Stavroula Bitsi^3^, Fiona B Ashford^4,5^, Ivan R Corrêa Jr^6^, David J Hodson^4,5^, Alejandra Tomas^3^, Giuseppe Deganutti^2^, Christopher A Reynolds^2,7^, Bryn M Owen^1^, Tricia M Tan^1^, James Minnion^1^, Ben Jones^1, #^, Stephen R Bloom^1^.

^1^ Section of Endocrinology and Investigative Medicine, Department of Metabolism, Digestion and Reproduction, Imperial College London, London, W12 0NN, United Kingdom.

^2^ Centre for Sport, Exercise and Life Sciences, Faculty of Health and Life Sciences, Coventry University, Alison Gingell Building, CV1 5FB, United Kingdom.

^3^ Section of Cell Biology and Functional Genomics, Department of Metabolism, Digestion and Reproduction, Imperial College London, London, W12 0NN, United Kingdom.

^4^ Institute of Metabolism and Systems Research (IMSR), and Centre of Membrane Proteins and Receptors (COMPARE), University of Birmingham, Birmingham, United Kingdom.

^5^ Centre for Endocrinology, Diabetes and Metabolism, Birmingham Health Partners, Birmingham, United Kingdom.

^6^ New England Biolabs, Ipswich, MA, USA.

^7^ School of Life Sciences, University of Essex, Wivenhoe Park, Colchester, CO4 3SQ, United Kingdom.

^#^ Corresponding author: Ben Jones ([ben.jones@imperial.ac.uk](mailto:ben.jones@imperial.ac.uk); +44 20 331 30348)

**Contents:**

- **Supplementary Figure 1**
- **Supplementary Figure 2**
- **Supplementary Figure 3**

**Supplementary Figure 1. Nanobit kinetic traces with GLP-1 and glucagon analogues.** (**A**) Recruitment of mini-G_s_ or β-arrestin-2 to GLP-1R-SmBiT in HEK293T cells, responses to GLP-1 and GLP-1-AIB2, *n*=5. (**B**) As for (A) but for GCGR-SmBiT and GCG, GCG-AIB2, GCG-H3 and GCG-AIB2H3. Mean ± SEM responses are shown.

**Supplementary Figure 2. Further data for SRB103 analogues.** (**A**) Assessment of DPP-4 sensitivity for each peptide by HPLC measurement of intact peptide remaining after incubation with recombinant DPP-4 at 37°C for 1 or 24 hours, *n*=3, compared by repeated measures two-way ANOVA with Tukey’s test; only SRB103Q *versus* SRB103H comparisons are shown. (**B**) Recruitment of mini-G_s_ or β-arrestin-2 to GLP-1R-SmBiT or GCGR-SmBiT in HEK293T cells, responses to SRB103 peptides, *n*=6. (**C**) Mini-G_s_, -G_i_, -G_q_, and β-arrestin-2 recruitment responses in HEK293T to 1 µM GLP-1, glucagon, SRB103H and SRB103Q, *n*=3-4. (**D**) Heatmap representation of data from (C) indicating vehicle-subtracted agonist-induced response after normalisation to the endogenous agonist (GLP-1 for GLP-1R, glucagon for GCGR). Mean ± SEM responses are shown.

**Supplementary Figure 3. SRB103 analogue responses in mice.** (**A**) Blood glucose results during intraperitoneal glucose tolerance tests (IPGTTs) performed in lean male C57Bl/6 mice (*n*=8-10/group) with 2 g/kg glucose injected IP at the same time as, 4 hours after, or 8 hours after 3 or 30 nmol/kg agonist injection. Timepoint and AUC comparisons both by repeated measures two-way ANOVA with Tukey’s test; only SRB103Q *versus* SRB103H comparisons are shown. (**B**) Blood glucose during insulin tolerance tests (0.5 or 1 U/kg actrapid insulin IP) performed 4 hours after administration of 10 nmol/kg agonist injection in lean male C57Bl/6 mice (*n*=8/group). Absolute and percentage reductions from 0 – 15 min are shown (the 0.75 u/kg absolute reduction relates to the data in Figure 5D) and compared by one-way ANOVA with Tukey’s test; only SRB103Q *versus* SRB103H comparisons are shown. (**C**) Plasma concentration of SRB103H and SRB103Q 4 hours after IP injection of 0.5 mg/kg agonist in lean male C57Bl/6 mice, as determined by radioimmunoassay, compared by two-tailed unpaired t-test. (**D**) Profiles of plasma concentration of SRB103H and SRB103Q after repeated blood sampling of lean male Sprague Dawley rats which received SC injection 4 mg/kg, as determined by radioimmunoassay, compared by two-way repeated measures ANOVA with Sidak’s test. * p<0.05 by indicated statistical test. Data are represented as mean ± SEM and with individual replicates where possible.
